# Using the entomological inoculation rate to assess the impact of vector control on malaria parasite transmission and elimination

**DOI:** 10.1186/1475-2875-9-122

**Published:** 2010-05-12

**Authors:** Ayesha M Shaukat, Joel G Breman, F Ellis McKenzie

**Affiliations:** 1 Division of International Epidemiology and Population Studies, Fogarty International Center, National Institutes of Health, Bethesda, MD, USA

## Abstract

**Background:**

Prior studies have shown that annual entomological inoculation rates (EIRs) must be reduced to less than one to substantially reduce the prevalence of malaria infection. In this study, EIR values were used to quantify the impact of insecticide-treated bed nets (ITNs), indoor residual spraying (IRS), and source reduction (SR) on malaria transmission. The analysis of EIR was extended through determining whether available vector control tools can ultimately eradicate malaria.

**Method:**

The analysis is based primarily on a review of all controlled studies that used ITN, IRS, and/or SR and reported their effects on the EIR. To compare EIRs between studies, the percent difference in EIR between the intervention and control groups was calculated.

**Results:**

Eight vector control intervention studies that measured EIR were found: four ITN studies, one IRS study, one SR study, and two studies with separate ITN and IRS intervention groups. In both the Tanzania study and the Solomon Islands study, one community received ITNs and one received IRS. In the second year of the Tanzania study, EIR was 90% lower in the ITN community and 93% lower in the IRS community, relative to the community without intervention; the ITN and IRS effects were not significantly different. In contrast, in the Solomon Islands study, EIR was 94% lower in the ITN community and 56% lower in the IRS community. The one SR study, in Dar es Salaam, reported a lower EIR reduction (47%) than the ITN and IRS studies. All of these vector control interventions reduced EIR, but none reduced it to zero.

**Conclusion:**

These studies indicate that current vector control methods alone cannot ultimately eradicate malaria because no intervention sustained an annual EIR less than one. While researchers develop new tools, integrated vector management may make the greatest impact on malaria transmission. There are many gaps in the entomological malaria literature and recommendations for future research are provided.

## Background

To fight malaria successfully, researchers must use current tools effectively and measure the impact of these tools precisely. This paper assesses the entomological inoculation rate (EIR) in relation to the major vector control interventions. EIR measures the intensity of malaria parasite transmission by anopheline vectors and vector control interventions are the only tools currently considered able to interrupt transmission. Vector control success against malaria is based on previous experiences in temperate climate countries where larval control and insecticide spraying of dwellings with dichlorodiphenyltrichloroethane (DDT) resulted in elimination of malaria from large areas of the globe [1,2]. EIR values are used to quantify the impact of insecticide-treated bed nets (ITNs), indoor residual spraying (IRS), and source reduction (SR) on malaria transmission. This analysis is extended by evaluating whether available vector control tools alone can ultimately eradicate malaria globally.

### Why the entomological inoculation rate (EIR)?

One can measure the intensity of malaria transmission several ways: Table 1 outlines some of these indices, highlighting their advantages and disadvantages [3-9]. Many of these indices, derived from field and theoretical data, are calculated using assumptions and they are generally not used for evaluating control programmes. The EIR remains the most direct measurement for assessing the effect of anti-vector actions because it quantifies the parasite-infected mosquito pool and its propensity to transmit infectious parasites to the human population.

**Table 1:** Major Indices of Malaria Transmission: Advantages and Disadvantages

Index	What is Measured	Advantages	Disadvantages
**Entomological Inoculation Rate (EIR)**	Infectious bites per unit time (usually per year)	Direct reflection of vector control and antigametocytocidal drugs	- No standard protocols - Variability in methodologies - Few trained specialists

**Parasite Rate (PR)**	Proportion of the population found to carry asexual parasites in RBCs; can also assess gametocyte rates; by age group	Direct reflection of inoculations, immunity, and treatment effectiveness in humans	- Microscopy "gold standard"; lacks sensitivity - Prone to technical efforts - Changes may occur following environmental and control factors

**Annual Parasite Index (API)**	Number of parasite infections in a well-defined geographical area; usually per 1,000 persons per year	Direct reflection of all prevention and control effects on humans	- Depends on active case detection system, which is often poor

**Spleen Rate (SR)**	Proportion of children 2-9 years of age with a palpable spleen	Non-invasive, indirect way of measuring impact of malaria on spleen	- Variability in examiners; many causes of splenomegaly - Point prevalence measurements can vary/change rapidly

### What is the EIR and how is it measured?

The EIR is the number of infectious bites per person per unit time, usually measured or expressed per year. It is the product of the human biting rate and the sporozoite rate:

The human biting rate (*Ma*) is the number of vectors biting an individual over a fixed period of time. *M *equals the number of *Anopheles *per person and *a *equals the average number of persons bitten by one *Anopheles *in one day. The sporozoite rate (*S*) is the fraction of vector mosquitoes present and biting that are considered infectious, i.e. *Anopheles *with sporozoites in their salivary glands [3,10]. Reducing any of these values would decrease the EIR. Several methods measure the human biting rate, including using "capturers" (human landing catches), pyrethrum spray catches, exit trap collections, and CDC light traps [11]. Many errors can emerge in estimating both the human biting rate and sporozoite rate. These result from variation in method used, attraction of mosquitoes to the capturer, and diligence of the technical teams [12].

**Figure 1:**
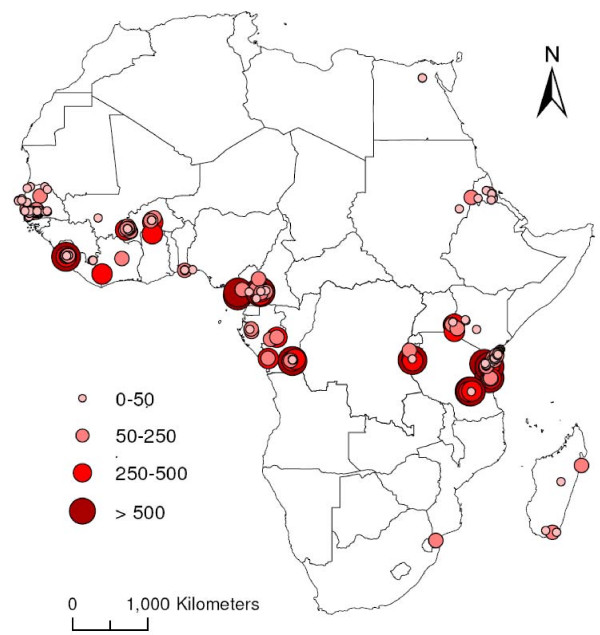
**Magnitude and geographical distribution of annual *Plasmodium falciparum *EIR estimates across Africa between 1980 and 2004**.

The 2009 review by Kelly-Hope and McKenzie of annual *P. falciparum *(A*Pf*) EIRs illustrates substantial gaps in the EIR data across Africa [13]. A*Pf *EIR estimates were available from only 23 of the 54 African countries, with 56% of the measures from four countries (Kenya, Burkina Faso, Tanzania, and The Gambia) [13]. Figure 1 shows that there can be huge variation in the EIR at the same geographic location, from village to country scale, even when seasonality of transmission is taken into account; for example, Tanzania shows an EIR variation of >10 times in the same area [13]. Lack of consistently used, standard EIR measurement methods means that two researchers may measure the EIR in the exact same location and time frame, yet calculate greatly different values.

Numerous factors influence the EIR, including temperature, altitude, rainfall, and urbanization [3]. In general, the EIR is directly proportional to temperature because heat accelerates the sporogonic cycle, the time necessary for ingested gametocytes to develop into infectious sporozoites. The optimal temperature for malaria transmission is 25-27°C and an average monthly relative humidity above 60% [14]. For the same reason, the EIR is inversely proportional to altitude because temperature decreases as altitude increases. The EIR is directly proportional to rainfall because female *Anopheles *mosquitoes lay their eggs in water. Generally, the EIR is inversely proportional to urbanization because with urbanization comes fewer bodies of water and greater pollution of water sources [15]. Therefore, tropical areas with warm temperature, heavy rainfall, high humidity, and efficient *Anopheles *vectors are ideal for malaria transmission [16]. These factors explain a large part of the variability in the EIRs across Africa.

An adult mosquito's lifespan is particularly important to transmit malaria. The mosquito must survive long enough for the parasite to complete sporogonic development from the point where gametocytes are ingested with the blood meal to the time when infectious sporozoites appear in the salivary glands. This process typically takes 10 days for *P. falciparum *[2]. Therefore, decreasing the lifespan of mosquitoes substantially decreases the EIR.

### Vector control interventions against malaria

Lacking widespread use of transmission-blocking drugs or vaccines, vector control remains the preferred strategy for reducing malaria transmission. It is the only available method "capable of bringing intense or moderate transmission down to the low levels where elimination is within reach" [17]. Insecticide-treated nets (ITNs), indoor residual spray (IRS), and source reduction (SR) are the major vector control tools. Certain tools are more appropriate depending on the mosquito's behaviour and environment. All interventions require careful planning, trained staff for implementation, rigorous supervision and evaluation, free or low-cost access and sustainability [18].

## Methods for evaluating the effect of vector control on EIRs

The analysis in this study is based primarily on a literature search using PubMed^®^, Web of Science^SM^, and Scopus™ for papers on EIR and ITN, IRS, or SR. Variations of these terms and different combinations were used when conducting the literature search. Once papers were found, the references were searched. Active researchers were contacted and asked about unpublished reports and past or on-going work. Studies were excluded if they did not have a control group for comparison with interventions or if they used methods other than ITN, IRS or SR. EIR was calculated as the product of the mean human biting rate multiplied by the mean sporozoite rate. The human biting rate and sporozoite rate were not reported separately in each study.

A total of eight vector control intervention studies were found that measured the EIR: four ITN studies [19-22], one IRS study [23], one SR study [24], and two studies that had separate ITN and IRS intervention groups [25,26]. All of the studies included a separate control group that did not receive the intervention, and the researchers took measurements from the control and the intervention groups at the same time. The studies reported different time frames for measuring the mean biting rate, either per night, month, season, or year. The different EIR values could not be directly compared, so percent differences in EIR were calculated between the intervention groups and the control groups to estimate the effects of the interventions. The Garki project was the only study that reported coverage values for intervention methods. Among all villages and rounds of the Garki project, coverage varied from 74% to 100%. In villages selected for follow up, coverage averaged 99% and varied, among villages and rounds, from 84% to 100%. The other seven studies did not report levels of coverage. Due to inadequate information in the reports, the studies were not weighted based on number of measurements, duration, or quality.

Three studies had EIR measurements for more than one year. For these studies, an average of the control group EIR and an average of the intervention group EIR data were calculated. These two averages were compared to calculate the percent difference in EIR between the control group and intervention group. The same method was used to analyse the data from the study that had two intervention groups and took EIR measurements for two years. In the two studies with ITN and IRS intervention groups, each group was compared to the control group and analysed separately.

## Results

### Insecticide-treated nets (ITN)

Six studies, performed in Tanzania, Kenya, and the Solomon Islands, measured the EIR with and without an ITN intervention. Table 2[19-22,25,26] summarizes these ITN studies. Two of the ITN studies collected EIR data for two consecutive years, Tanzania from 1995-1996 [25] and Kenya from 1990-1991 [22]. Both of these studies saw a significantly greater decrease in EIR between the ITN and control villages in the second year (90% lower in Tanzania and 75% lower in Kenya) compared to that in the first year (42% higher in Tanzania and 55% lower in Kenya). Interestingly, the first year of the Tanzania study showed an increase in the ITN group's EIR compared to the control group. Increased compliance from the community during the second year, or perhaps greater experience with treating the nets and implementing the intervention, serve as possible explanations for greater EIR reduction in the second year of the studies.

**Table 2:** EIR Studies Tied to Insecticide Treated Bed Net Intervention

Location/Year Study Done/Reference	Insecticide	Mosquito	Method	EIR	Parasite Rate
Tanzania: highland hamlets (altitudes 784 - 1148 m) and lowland hamlets (199-300 m) October 1998-August 2000 [20]	[0.02] g alphacypermethrin/m^2^ nets not re-treated during study	*An. gambiae s.l., An. funestus *and *An.marshallii s.l.*	light traps, pyrethrum spray, window exit traps and ELISA	Infectious bites/person/year Highland: Control = 10.4 ITN = 3.2 Lowland: Control = 148.6 ITN = 37.5 Highland = **69% Reduction** Lowland = **75% Reduction**	Highlands: 6 months-2 years: Control = 54.1% ITN = 31.4% 2-5 years: Control = 73% ITN = 44.3% 6-12 years: Control = 67.7% ITN = 49.4% Lowlands: 6 months-2 years: Control = 82.9% ITN = 63.1% 2-5 years: Control = 88.8% ITN = 78.3% 6-12 years: Control = 83.3% ITN = 80.6% Highlands = **36% Reduction** Lowlands = **13% Reduction**

Lake Victoria shore in Western Kenya January 1997-February 2000 [19]	[0.5] g permethrin/m^2^ nets re-treated every 6-11 months	*An. gambiae *and *An. funestus*	pyrethrum spray sheet collection and ELISA	Infectious bite/person/month Control = 0.93 ITN = 0.08 **91% Reduction**	**Not reported**

North East Tanzania 1997-1998 [21]	[0.02] g alphacypermethrin/m^2 ^or [0.1] g lambdacyhalothrin/m^2^	*An. gambiae s.l., An. funestus, An. marshallii *and *cx. quinquefasciatus*	light traps and ELISA	Infectious bites/person/night Control = 3.24 alphacypermethrin = 0.153 lambdacyhalothrin = 0.140 alphacypermethrin = **95% Reduction** lambdacyhalothrin = **97% Reduction**	Rates of re-infection with asexual malaria parasites after treatment with chlorproguanil-dapsone: Control = 30.8% alphacypermethrin = 8.0% lambdacyhalothrin = 7.5% alphacypermethrin = **74% Reduction** lambdacyhalothrin = **76% Reduction**

North-east Tanzania 1995-1996 [25]	[0.01] g lambdacyhalothrin/m^2 ^for two villages [0.02] g lambdacyhalothrin/m^2 ^for two villages nets re-treated after 7 months	*An. gambiae *and *An. Funestsus*	light traps, window exit traps, pyrethrum spray collection and ELISA	Infectious bites/person/night 1995: Control = 1.04 ITN = 1.48 1996: Control = 0.773 ITN = 0.08 1995 = **42% Increase** 1996 = **90%Reduction**	**Not reported**

Western Kenya March-June 1990 and 1991 (high transmission season) [22]	[0.5] g permethrin/m^2^ nets re-treated October 1990	*An. Gambiae s.s.*	night biting collections and ELISA	Infectious bites/person/night 1990 high transmission season: Control = 0.47 ITN = 0.21 1991 high transmission season: Control = 0.36 ITN = 0.09 1990 = **55% Reduction** 1991 = **75% Reduction**	Incidence of Plasmodium *falciparum *parasitemia ≥ 2,500/mm^2 ^in children less than six years old 1990 high transmission season: Control = 135 (94) ITN = 77 (53) 1991 high transmission season: Control = 64(82) ITN = 51(64) 1990 = **43% Reduction** 1991 = **20% Reduction**

Northern Guadalcanal, Solomon Islands November 1987-June 1988 [26]	[0.5] g permethrin/m^2^ nets re-treated August 1987	*An. farauti *and *An. Puctulatus*	Human landing catch and ELISA	Infectious bites/person/night Control = 2.204 ITN = 0.129 **94% Reduction**	*P. falciparum* Control = 29% ITN = 21% *P. vivax* Control = 12% ITN = 14% *P. falciparum *= **28% Reduction** *P. vivax *= **17% Increase**

### Indoor residual spraying (IRS)

Table 3[23,25,26] summarizes the three studies that used IRS and measured the impact on the EIR. Both the Tanzania [25] and Solomon Islands [26] studies had one community that received ITNs and one that received IRS (in addition to the control community). These studies are particularly valuable because they measure the impact of two vector control methods on the EIR. The areas given ITNs and IRS had similar populations and locations, allowing direct comparison of the impact of the two interventions. In the second year of the Tanzania study, ITNs caused a 90% decrease in EIR and IRS a 93% decrease, both highly significant; the ITN and IRS effects did not differ. The Solomon Islands study had a significantly greater EIR reduction in the ITN areas, 94% relative to the control, compared to the IRS areas, 56%. These were the only two studies found that directly compared the impact of ITN and IRS on the EIR in the same place at the same time. Therefore, there is not enough evidence to generalize and determine which intervention has the greatest effect on the EIR.

**Table 3:** EIR Studies Tied to Indoor Residual Spray Intervention

Location/Year Study Done/Reference	Insecticide	Mosquito	Method	EIR	Parasite Rate
North-east Tanzania 1995-1996 [25]	[0.03] g lambdacyhalothrin/m^2^ re-sprayed 7-8 months after initial spray	*An. gambiae *and *An. funestsus*	light traps, window exit traps, pyrethrum spray collection and ELISA	Infectious bites/person/night 1995: Control = 1.04 IRS = 0.98 1996: Control = 0.773 IRS = 0.057 1995 = **5.7% Reduction** 1996 = **93% Reduction**	**Not reported**

Northern Guadalcanal, Solomon Islands November 1987-June 1988 [26]	[2] g DDT/m^2^	*An. farauti *and *An. puctulatus*	Human landing catch and ELISA	Infectious bites/person/night Control = 2.204 IRS = 0.9675 **56% Reduction**	*P. falciparum* Control = 29% IRS = 46% *P. Vivax* Control = 12% IRS = 9% *P. falciparum *= **59% Increase** *P. vivax *= **25% Reduction**

Garki, Nigeria September 1969-February 1976 [23]	[2] g propoxur/m^2^ re-sprayed every 2 months	*An. Gambiae s.l. *and *An. funestus*	Human landing collection, pyrethrum spray collection, exit trap collection, outdoor resting collection and ELISA	Infectious bites/person/wet season (wet season 1972: May 22-Oct. 22 1973: June 18-Nov. 4) Control: Village 1: 1972 = 17 1973 = 21 Village 2: 1972 = 25 1973 = 28 IRS: Village 3: 1972 = 0 1973 = 10 Village 4: 1972 = 3 1973 = 4 1972 = **93% Reduction** 1973 = **71.4%Reduction**	*P. falciparum* Control 1972 = 43.3% 1973 = 47.5% IRS 1972 = 36.8% 1973 = 35.0% *P. malariae* Control 1972 = 13.0% 1973 = 11.19% IRS 1972 = 13.3% 1973 = 13.3% *P. falciparum* 1972 = **15% Reduction** 1973 = **26% Reduction** *P. malariae* 1972 = **2.3% Increase** 1973 = **19% Increase**

### Source reduction (larval control)

Only one study, in Dar es Salaam, measured EIR differences tied to source reduction. The control area had an annual EIR of 1.06 (0.64-1.77) and the area that received the microbial larvicide *Bacillus thuringiensis israeliensis *(*Bti*) had an annual EIR of 0.56 (0.43-0.77). There was a lower EIR reduction (47%) due to SR compared to the ITN and IRS studies [24]. However, SR is likely to be particularly effective in urban areas, where breeding places are man-made and can be identified, mapped, and treated [27]. SR should receive increasing attention for malaria control because more than half of the African population is expected to live in urban and peri-urban areas by 2030 [24].

## EIR analysis

In each of these eight studies, the malaria vector population size and sporozoite rates decreased, causing a great decrease in the EIR. The studies used different time frames to measure EIR; each study reported the defined EIR as infective bites per person - per year, season, month, or night.

One approach to analysing the data would be to standardize all the EIRs to one year. For example, in Molineaux's and Gramiccia's Garki project data, in 1973 one village that received ITNs had 28 infectious bites per person per wet season. The wet season in 1973 lasted 139 days [23]. Therefore, if the EIR were calculated for the entire year, it would be 73.6 infectious bites per person per year. However, EIR is highest during the rainy season. If EIRs taken during the rainy season were used and if these values were extended to represent the entire year, the calculations would grossly overestimate the annual EIR. Additionally, the EIRs between studies could not be directly compared because the study areas have different climate, altitude, and population density.

**Figure 2:**
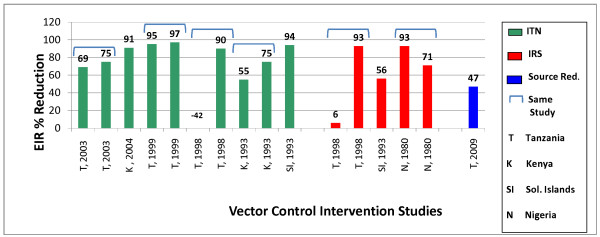
**Entomological inoculation rate percent reduction by vector control intervention**.

To avoid this problem, EIR percent changes based on values with and without the implementation of vector control interventions were calculated. Figure 2 summarizes the EIR percent reduction data from the eight studies in this review. All the studies that lasted for more than one year, aside from the Garki project, had a greater reduction in the EIR the second year. The 1998, Tanzania study had a higher EIR value in the intervention group than the control group and it is an outlier from the rest of the data [25]. The other studies showed a large percent EIR reduction in their vector control intervention groups, however none reached 100% reduction.

The EIRs calculated in this study were compared to the report by Beier *et al*, which relates EIRs to parasite rates [28]. Beier *et al *reported that only annual EIRs less than one could reduce parasite rates to levels that could interrupt malaria transmission. They concluded that it may not be possible to achieve dramatic decreases in the prevalence of *P. falciparum *infection at sites in Africa, unless control measures sustainably reduce EIRs to levels well below one infective bite per person per year [28].

**Figure 3:**
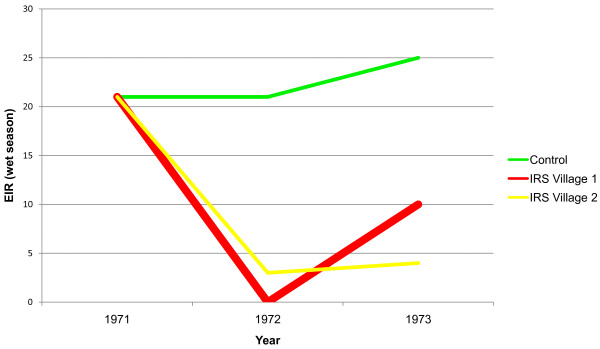
**Entomological inoculation rates following insecticide residual spraying in Garki, Nigeria, 1971-1972**.

Putting aside previously mentioned concerns about extending the EIRs measured per night, month, or season to represent the entire year, the annual EIR for all the studies in this review were calculated to relate these studies to the findings of Beier *et al*. Of all eight studies, only the Garki project using IRS with propoxur, a carbamate insecticide, reduced the annual EIR to less than one infective bite per person (Figure 3) [23]. However, the Garki project interventions only caused temporary reduction of the EIR. In 1972, the EIR was reduced to zero, but despite re-spraying houses every two months, the EIR increased to ten the following year [23]. Therefore, the only intervention study that decreased the EIR to <1 did not maintain such a level for more than one year. Based on these data, current vector control methods are not enough to eradicate malaria. ITNs, IRS, and SR are imperfect, but researchers must continue using and improving them.

### Integrated vector management (IVM)

Malaria researchers believe that IVM is an effective strategy because it uses two or more vector control methods, with each method targeting a setting most susceptible to that intervention. Although IRS, ITN, and SR are all effective individually, they complement each other and have a synergistic impact when used together [29]. IVM involves a "rational decision-making process for the optimal use of resources for vector control" [30]. It requires reconsidering the combination of vector control methods over time, as the environment, epidemiology, and resources change. IVM is not limited to controlling malaria. In 2004, the World Health Organization recommended IVM globally for the control of all vector-borne diseases [30].

IVM programmes have brought great reductions in malaria transmission. Killeen *et al *created a mathematical model to predict the impact of IVM on EIR. Their model predicts a 15- to 25-fold reduction in EIR when using ITNs and larval control. Their model predicts that ITNs and IRS are the most effective tools available for reducing the EIR; source reduction amplifies the results. Despite their dramatic predicted impact on the EIR, the simulated integrated control programmes in the model by Killeen *et al *failed to reduce the EIR to less than one at any of their sites [5].

**Figure 4:**
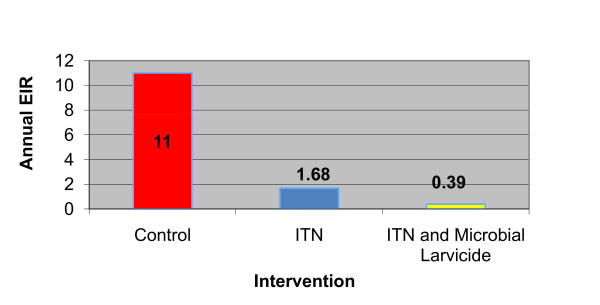
**The study by Fillinger *et al *integrated vector management in Western Highlands, Kenya entomological inoculation rate**. Reported Annual Entomological Inoculation Rates for Control, Insecticide Treated Bed Net, and Integrated Vector Management Groups

The work of Fillinger *et al *[31] in the western highlands of Kenya is the first study that measures the impact of an IVM approach directed at both larval and adult mosquitoes. Their study's control groups had EIRs of 10-12 infectious bites per person per year. With ITNs, the annual EIR dropped to 1.68 infectious bites per person. Once the researchers added microbial larvicide to ITNs, the annual EIR dropped to 0.39 infectious bites per person, a 73% reduction [31]. Therefore, using ITNs and SR together reduced the annual EIR to less than one (Figure 4). The parasite prevalence was similar in all the groups at baseline measurements. There was a significantly lower prevalence of new infections in the intervention group that received larvicide (7.0%, 95% confidence interval 4.6%-10.7%) compared to the group that did not receive larvicide (12.8%, 95% confidence interval 9.7%-15.9%).

These studies illustrate that an integrated vector approach can meaningfully reduce EIR and that larval control amplifies the effect of adult vector control. Vector control interventions do not sustainably decrease EIR values low enough to effectively drive down malaria prevalence. However, IVM organizes these tools to make the greatest impact on malaria transmission, while researchers may invest time in developing new methods to fight the disease.

## Conclusions

### Conclusions and future research directions

**Figure 5:**
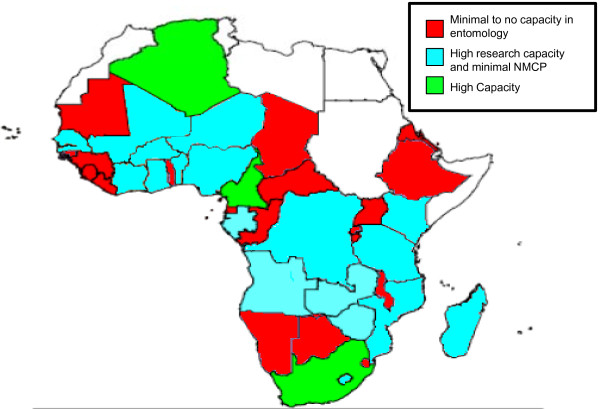
**Vector surveillance capacities in National Malaria Control Programmes (NMCP) and research institutions in 38 African Countries**. Adapted from African Network on Vector Resistance: ANVR Newsletter Issue No. 1, 2006

This is the first review that links vector control methods to the EIR. The results illustrate that for areas with intense transmission, current vector control tools are not enough to eradicate malaria. Use of ITNs, IRS, or SR individually will not decrease the annual EIR low enough to assure consistently significant and sustained reductions in malaria parasite prevalence. This analysis is based on the assumption that an EIR below one is needed to interrupt malaria transmission, though caution is required when following this assumption. Through conducting this review, gaps were found in the literature and several recommendations are included:

• Researchers should conduct more EIR studies in relation to vector control interventions; there are only nine studies that measure the impact of vector control interventions on the EIR.

• Researchers must standardize EIR methods to allow comparisons between studies. EIR percent changes were calculated for comparisons of different studies. If researchers agreed on a standard EIR time frame measurement, then individuals could directly compare different studies.

• Researchers should include background information about communities in their studies. The EIR is influenced by factors such as altitude, population density, rainfall, socio-cultural attitudes and behaviour, as well as current malaria control actions and their impact on human infection and disease. Additionally, researchers should include information about the coverage in intervention groups. Among the studies in this paper, the Garki project was the only study that reported level of coverage. These details are often missing from articles on EIR studies and they are essential for comparing different studies.

• It is necessary to conduct long term (>1 year) studies to measure the sustainability of the intervention's impact. If researchers had conducted the Garki project for only one year, they would have a false sense of confidence in the impact of IRS for their site.

• Increased research in source reduction and integrated vector management studies are strongly advised, particularly for urban, peri-urban, and epidemic-prone areas. Integrated vector management provides an approach that makes the most use out of existing tools. Only two studies were found that used ITNs and SR, one of which was a theoretical analysis [5,31]. Researchers should conduct an IVM study with all three vector control interventions to measure their additive or synergistic effect.

• Measurement of the EIR should also be coordinated with parasitological, clinical, and meteorological indices, assessed at the same time and in the same place to reflect the most accurate picture of transmission. These factors are interrelated, however, researchers have yet to conduct a study that takes all these factors into account.

• There must be a focus on building entomological operational and research capacity. Figure 5 illustrates vector surveillance capacities in national malaria control programmes and research institutions in 38 African countries. Only South Africa, Algeria, and Cameroon have a high entomology capacity [32]. This figure is based on data from 2006, so there may have been improvements since then. The President's Malaria Initiative (PMI), the Global Fund for AIDS, Tuberculosis and Malaria, and other public health programmes have made major investments in vector control interventions, yet we lack trained researchers to conduct the work. Accomplishing the goals defined by these ambitious programmes requires a highly skilled, supervised, and supported team of entomology and vector control specialists in addition to a large cadre of scientists, public health, and operational specialists in all malaria disciplines.

When drawing conclusions from EIR studies, one must keep in mind the difficulty in measuring EIR. Many factors influence EIR, including location, time of year, and measurement method. Additionally, EIR studies may not measure the full impact of a particular intervention because vector control measures have increased impact over time. Although measuring EIR can be difficult, expensive, and time consuming, it remains the most direct measurement for assessing the effect of vector control interventions.

## Competing interests

The authors declare that they have no competing interests.

## Authors' contributions

AMS identified data sources, carried out data analysis, and wrote the first draft of the manuscript. JGB conceived the idea for the research and contributed significantly to formatting and editing the manuscript. FEM critically revised the manuscript. All authors read and approved the final manuscript.
